# Editorial: Genetics and multi-omics approach in metabolic liver disorders

**DOI:** 10.3389/fendo.2025.1620522

**Published:** 2025-05-27

**Authors:** Omar Ramos-Lopez, Prabu Paramasivam, Gautam K. Pandey, Sathishkumar Chandrakumar

**Affiliations:** ^1^ Medicine and Psychology School, Autonomous University of Baja California, Tijuana, Baja California, Mexico; ^2^ Department of Pharmaceutical Sciences, College of Pharmacy, University of New Mexico Health Sciences, Albuquerque, NM, United States; ^3^ Department of Genetics, University of North Carolina, Chapel Hill, NC, United States; ^4^ Department of Ophthalmology, University of California, Los Angeles, Los Angeles, CA, United States; ^5^ Doheny Eye Institute, Pasadena, CA, United States

**Keywords:** liver disorders, NAFLD/MASLD, genetics, multi-omics, bioinformatics, precision medicine

Liver diseases are major causes of morbidity and mortality in developing and developed countries, accounting for millions of deaths annually attributable to organic and systemic complications (1). In addition to viral hepatitis and alcohol abuse, nonalcoholic fatty liver disease (NAFLD), more recently renamed metabolic dysfunction-associated steatotic liver disease (MASLD), is one of the most common causes of chronic liver disease worldwide; it is closely linked to obesity, metabolic syndrome, and type 2 diabetes (2). Lipid metabolism disorders, insulin resistance, and dysregulation of cytokines/adipokines are implicated in the development of NAFLD/MASLD, also contributing to its evolution from steatosis to steatohepatitis, cirrhosis, and even hepatocellular carcinoma (3).

The pathogenesis of NAFLD/MASLD and other liver diseases is influenced by both genetic and environmental factors. In this regard, genomic methodologies, including genome-wide association studies (GWAS) and candidate-gene approaches, have identified polymorphisms and other structural DNA variants associated directly with the occurrence of NAFLD/MASLD or related metabolic traits, including abdominal obesity and glucose and lipid disorders (4). Furthermore, multi-omics technologies, including transcriptomics, proteomics, lipidomics, glycomics, and metabolomics, have allowed the characterization of targets and pathways involved in the pathophysiology of liver metabolic diseases (5). This Research Topic includes five original articles and one systematic review exploring the role of genetics and related multi-omics features involved in the onset and progression of metabolic liver disorders in order to envisage innovative precision medicine interventions.

Mendelian randomization (MR) is a method that applies genetic variation data to evaluate causal relationships between potentially modifiable risk factors and health outcomes. In this context, Song et al. conducted MR analyses managing the GWAS dataset to dissect associations between air pollutants, NAFLD/MASLD, and liver function indicators in Europeans. Although they found no robust evidence of causal relationships between air pollution and NAFLD/MASLD, a relevant association was identified between the air pollutant PM10 and serum albumin levels, suggesting that this chemical may affect protein metabolism in the liver. Using a similar MR approach, Liu et al. identified a causal association between inflammatory cytokines (including RANTES, IL-2, MIF, TRAIL, and SCF) and NAFLD/MASLD, with a small proportion of the effect being modestly mediated by the serum levels of ferritin. Moreover, Zheng et al. performed MR analysis to infer a genetically determined causal connection between plasma lipidomes (namely triacylglycerol and phosphatidylcholine) and NAFLD/MASLD, where specific plasma metabolomes (i.e., Ximenoylcarnitine, caffeine-to-paraxanthine ratio, pregnenetriol sulfate, 1-palmitoy1-2-oleoyl-GPC, and glucose to N-palmitoyl-sphingosine ratio) acted as potential mediators.

Multi-omics approaches have been applied in biomedical research to holistically elucidate the mechanisms of action of various drugs for the treatment of liver diseases as well as to improve their diagnosis and clinical management, with appropriate bioinformatics and analytical tools. In this regard, Jiang et al. integrated proteomic and metabolomic information to explore immunological and molecular biomarkers for clinical diagnosis and differentiation of occult hepatitis B infection (OBI) and HBsAg-positive hepatitis B virus (HBV) infection. For this purpose, they constructed a molecule-based diagnostic model with acceptable classification accuracy that effectively was able to differentiate HBsAg-positive and OBI groups, thus representing a non-invasive tool for characterizing specific phenotypes in these diseases. In addition, Wang et al. explored the multi-target mechanism of dapagliflozin on mitigating diabetic liver injury in mice based on bioinformatics testing and experimental verification, including *in vivo* and *in vitro* assays. They found that dapagliflozin ameliorated diabetic liver injury by inhibiting the ERK/IKKβ/NF-κB signaling pathway, decreasing lipid deposition and blood glucose, oxidative stress levels, and the inflammatory and apoptosis-related proteins and mRNA levels. Furthermore, due to the need to identify and validate more accurate molecular markers to effectively predict NAFLD/MASLD disease and its progression, Shen et al. conducted a systematic review and meta-analysis to confirm the relevance between circulating irisin levels and NAFLD/MASLD pathogenesis. The results revealed that circulating irisin levels were significantly lower in NAFLD/MASLD patients than in healthy controls, with relevant impacts on disease detection and pharmacological treatment.

In summary, the articles published in this Research Topic contribute to a better understanding of the pathophysiological processes underlying liver metabolic diseases, primarily but not limited to NAFLD/MASLD. These findings also provide guidance on the relevance of biomarkers and therapeutic targets for the design and implementation of intervention strategies under a precision medicine approach, integrating knowledge from diverse omics areas and environmental factors involved in the development of these diseases. The use of bioinformatics, machine learning, and other artificial intelligence tools is essential to harmonize heterogeneous data from omics technologies for translation in clinical practice.
